# Low-dose dexamethasone as a treatment for women with heavy menstrual bleeding: protocol for response-adaptive randomised placebo-controlled dose-finding parallel group trial (DexFEM)

**DOI:** 10.1136/bmjopen-2014-006837

**Published:** 2015-01-14

**Authors:** P Warner, C J Weir, C H Hansen, A Douglas, M Madhra, S G Hillier, P T K Saunders, J P Iredale, S Semple, B R Walker, H O D Critchley

**Affiliations:** 1 Centre for Population Health Sciences, University of Edinburgh, Edinburgh, UK; 2 Edinburgh Health Services Research Unit, Edinburgh, UK; 3 MRC Tropical Epidemiology Group, London School of Hygiene and Tropical Medicine, London, UK; 4 MRC Centre for Reproductive Health, University of Edinburgh, Edinburgh, UK; 5 MRC Centre for Inflammation Research, University of Edinburgh, Edinburgh, UK; 6 Clinical Research Imaging Centre, University of Edinburgh, Edinburgh, UK; 7 British Heart Foundation Centre for Cardiovascular Science, University of Edinburgh, Edinburgh, UK

**Keywords:** STATISTICS & RESEARCH METHODS

## Abstract

**Introduction:**

Heavy menstrual bleeding (HMB) diminishes individual quality-of-life and poses substantial societal burden. In HMB endometrium, inactivation of cortisol (by enzyme 11β hydroxysteroid dehydrogenase type 2 (11βHSD2)), may cause local endometrial glucocorticoid deficiency and hence increased angiogenesis and impaired vasoconstriction. We propose that ‘rescue’ of luteal phase endometrial glucocorticoid deficiency could reduce menstrual bleeding.

**Methods and analysis:**

DexFEM is a double-blind response-adaptive parallel-group placebo-controlled trial in women with HMB (108 to be randomised), with active treatment the potent oral synthetic glucocorticoid dexamethasone, which is relatively resistant to 11βHSD2 inactivation. Participants will be aged over 18 years, with mean measured menstrual blood loss (MBL) for two screening cycles ≥50 mL. The primary outcome is reduction in MBL from screening. Secondary end points are questionnaire assessments of treatment effect and acceptability. Treatment will be for 5 days in the mid-luteal phases of three treatment menstrual cycles. Six doses of low-dose dexamethasone (ranging from 0.2 to 0.9 mg twice daily) will be compared with placebo, to ascertain optimal dose, and whether this has advantage over placebo. Statistical efficiency is maximised by allowing randomisation probabilities to ‘adapt’ at five points during enrolment phase, based on the response data available so far, to favour doses expected to provide greatest additional information on the dose–response. Bayesian Normal Dynamic Linear Modelling, with baseline MBL included as covariate, will determine optimal dose (re reduction in MBL). Secondary end points will be analysed using generalised dynamic linear models. For each dose for all end points, a 95% credible interval will be calculated for effect versus placebo.

**Ethics and dissemination:**

Dexamethasone is widely used and hence well-characterised safety-wise. Ethical approval has been obtained from Scotland A Research Ethics Committee (12/SS/0147). Trial findings will be disseminated via open-access peer-reviewed publications, conferences, clinical networks, public lectures, and our websites.

**Trial registration number:**

ClinicalTrials.gov NCT01769820; EudractCT 2012-003405-98.

## Introduction

### Background

Heavy menstrual bleeding (HMB) is defined as excessive menstrual blood loss that interferes with the woman's physical, emotional, social or material quality-of-life.^1^ The prevalence of excessive menstrual bleeding in developing countries is reported as 4–9%.^2^ Community surveys of UK menstruating women have found 35–52% prevalence of reporting ‘heavy periods’ in the past 6 months,^3^ ^4^ and 25% annual cumulative incidence of reporting periods as ‘heavy’,^4^ but with respect to putative HMB, only 15% who report both heavy periods *and* that their periods are ‘a marked/severe problem’.^3^ Annually, 1 million UK women seek help for HMB,^1^ and an estimated 3.5 million work days are lost.^5^ Conservative estimates of annual direct and indirect economic costs of menstrual bleeding problems in the USA are US$1 billion and US$12 billion, respectively (in year 2005$).^6^

Surgical treatments for HMB (hysterectomy, endometrial ablation) end fertility, and hysterectomy is high-cost major surgery. Among those aged 30–40 years, uterine fibroids are often the cause of HMB,^7^ frequently necessitating surgery. In the US 10–15% of women aged 25–64 have hysterectomy for fibroids^8^ costing $3 billion annually.^6^ Hysterectomy remains a common intervention even in the absence of large fibroids.^9^ It is estimated in England and Wales, that annually about 80 000 women are referred for the first time to hospital with HMB and approximately 28 000 (35%) undergo surgical treatment.^10^ A national 4-year audit has reported that in the year following first attendance at hospital for HMB, 43% of women received surgery (8183 followed up).^11^ However, given half of all UK-born babies (47%) are to women aged 30 or older,^12^ fertility-ending surgery is not always acceptable.

Medical therapy for HMB is either ineffective,^10^ or associated with unacceptable side effects. The Levonorgestrel intrauterine system (LNG-IUS), a hormonal contraceptive now licensed as treatment for HMB, is unsuitable for women seeking to become pregnant. LNG-IUS can cause amenorrhoea, or for other users there is ongoing and unpredictable unscheduled bleeding, and these consequences can be unacceptable to women.^13^ The audit reported that in the first year after attendance for HMB, oral medication and IUS were received by 29% and 33%, respectively, but these were the ‘final’ treatment for only 12% (over half switched from oral medication) and 22% (one third switched from IUS).^11^ IUS and systemic progestin therapies for HMB are discontinued by up to one in five users due to side effects.^14^ A recent meta-analysis concludes that LNG-IUS is less cost-effective than hysterectomy for HMB.^15^ ^16^

HMB often occurs in combination with other symptoms.^17^ ^18^ The audit 1-year follow-up found only 35% of women were at least ‘satisfied’ at the prospect of current menstrual symptoms continuing, as *currently* experienced, for the next 5 years.^11^ There is therefore unmet need for cost-effective and acceptable therapy for HMB, particularly a medical therapy which preserves fertility and is compatible with becoming pregnant while on treatment.

### Mechanistic rationale for intervention

The cause(s) of HMB are not well understood. Approximately 48% of cases of HMB referred to secondary care occur in the absence of obvious pathology.^19^ In the normal menstrual cycle, blood vessel proliferation, differentiation and vasoconstriction in the endometrium is tightly regulated to ensure that a controlled and self-limited endometrial shedding occurs at menses. This is followed by a self-limiting inflammatory response to endometrial injury to ensure successful healing with a return to normal architecture, prior to the next cycle of vascular proliferation.^20^ These cyclic processes are orchestrated by dynamic changes in sex steroids and their interplay with endocrine, vascular and immune systems. Perimenstrual disturbance in local molecular and cellular mechanisms that are likely to lead to heavy and/or prolonged bleeding, include: (1) decreased vasoconstriction; (2) decreased vascular homeostasis; (3) an excessive inflammatory response at menses; and (4) defective repair of the postmenstrual endometrium. Deficient vascular development and abnormal angiogenesis have been reported in women with HMB.^20^

Glucocorticoids promote vasoconstriction and inhibit angiogenesis, so HMB could result from local endometrial glucocorticoid deficiency.^21–23^ We have shown that endometrium from women with HMB has increased expression of 11β hydroxysteroid dehydrogenase type 2 (11βHSD2), an enzyme which inactivates the major endogenous glucocorticoid, cortisol. This may cause local deficiency in endometrial cortisol,^21^ and result in an inadequate hypoxic signal at the time of progesterone withdrawal.^20^

We propose a novel therapy with synthetic glucocorticoid to ‘rescue’ luteal phase endometrial deficiency of cortisol in women with HMB, ensuring endometrial vascular differentiation and inflammation are properly controlled during the peri(menstrual) time of the cycle. There are several potential approaches, including use of a glucocorticoid receptor (GR) agonist which is less susceptible than cortisol to inactivation by 11βHSD2. Dexamethasone has higher affinity for GR than cortisol, but lower affinity for 11βHSD2. Therefore, our proposed treatment is a new therapeutic use of an existing, well-characterised medical treatment—oral dexamethasone—administered at a low ‘replacement’ dose.

### Objectives

The objectives for this trial of oral dexamethasone for amelioration of HMB, are to: (1) identify the efficacy of oral dexamethasone, and optimal dose to use; (2) gather safety data; (3) gather methodological and mechanistic insight to allow further development of this or similar treatment option.

## Design and methods

### Trial design

A response-adaptive parallel group randomised-controlled trial was proposed, comparing oral dexamethasone (in a range of doses) with placebo treatment, over three menstrual cycles. This allows, at intervals across enrolment, adaptation of the investigational treatment allocation probabilities, in response to the outcome data already collected, so those subsequently enrolled are more likely to be randomised to doses that are more informative about the dose–response relationship. This design ensures as robust as possible identification of the optimal dose, and maximal study power to estimate the effect of dexamethasone versus placebo, assuming treatment for three menstrual cycles. Objective measurement menstrual blood loss (MBL) was selected as the outcome, assessing treatment effect in terms of reduction from baseline in MBL (in the 2nd and 3rd of these treated cycles). This outcome is well suited to the adaptive design context since MBL data are available promptly after treatment, to inform adaptation. An adaptive design has efficiency and ethical advantages. Efficiency gains in terms of sample size required are in the range 25–40% for a broad spectrum of adaptive designs.^24^ This efficiency itself constitutes an ethical advantage,^25^ and additionally such a design ensures that more women are randomised to more effective doses (see online supplementary figure).

Development of a Bayesian adaptive design requires extensive preliminary simulation studies to explore empirically the performance of candidate designs.^24^ ^26^ Simulations were performed via fractional factorial design, covering a range of design options and model assumptions, including: proportion allocated to placebo (constant throughout); number of active doses; variance of the primary outcome (change in MBL from baseline); shape of the true dexamethasone dose–response curve for the primary outcome; accrual rate of patients to the study; and specific features of the adaptation process, including the method determining how the randomisation schedule would be adapted and the frequency with which this takes place. The simulations allowed the frequentist properties (statistical power and significance level) of candidate adaptive designs to be assessed. The final design selected is one which performs well across a broad range of scenarios. The adaptive design development process will be reported in detail elsewhere *(CH et al, manuscript in preparation*).

On the basis of the simulations, the specific features of the design selected were: 29% of patients allocated to placebo throughout; six active doses (0.4–1.8 mg total daily divided dose of dexamethasone) starting with equal allocation probability; and five adaptations, evenly spaced after 16, 32, 50, 66 and 84 randomisations (see online supplementary figure).

### Study setting

The participant population will be women reporting HMB who are referred for management to gynaecology outpatient departments in NHS Lothian (NHSL), Scotland, or who attend a community gynaecology clinic (self-referral or general practitioner (GP) referral). Additionally, through the Scottish Primary Care Research Network (SPCRN), women on SPCRN-participating Lothian GP practice lists who are potentially eligible (ie, who have codes on their GP practice system suggestive of HMB), will be sent brief information about the study. Any of these women who go on to contact the study team will be invited to be assessed for eligibility at an initial appointment at an NHSL clinic.

### Participants and recruitment

The entry criteria are listed in box 1. Women with symptoms of HMB will be given full information about the study and allowed ample time to read the information and consider whether they wish to participate. Women who fulfil the first four inclusion criteria, do not fulfil any exclusion criteria, and agree to participate, will be invited to undergo MBL screening to confirm they fulfil the fifth inclusion criterion. Women who decline participation or are ineligible will be offered routine NHSL gynaecological care.

Box 1 **Entry criteria**

Inclusion criteria

- Problem of heavy menstrual bleeding regardless of the presence or not of fibroids
- Age 18 years and over
- Premenopausal, describing menstrual cycles every 21–42 days
- Able to comply with study-related procedures/assessments
- Average measured blood loss (MBL), over two screening menstrual cycles, greater/equal to 50 mL

Exclusion criteria

- Currently breast feeding
- Known coagulation disorder
- Renal or liver dysfunction
- Ongoing thyroid dysfunction*
- Diabetes mellitus
- Hereditary galactose intolerance, lactase deficiency or glucose galactose malabsorption†
- History or current uterus, cervix, ovarian or breast cancer
- Pharmacologically treated moderate/severe hypertension
- Psychotic depressive illness
- Alcohol or drug abuse
- Mental capacity rendering her unable to understand the nature and scope of the study
- Participation in treatment phase in any earlier DexFEM work-up study
- Currently enrolled in an investigational drug or device study or participated in such a study within the previous 30 days and is still in exclusion period
- Pregnancy possible during the period of study participation‡
- Needing to, or intending to, continue taking any of the following *prohibited medications*§:
  Warfarin
  Sex steroid administration by any route
  Acetylsalicylic acid (aspirin)
  Mefenamic acid
  Antifibrinolytic drugs such as tranexamic acid
  Gonadotropin-releasing hormone agonist and antagonist¶
  Glucocorticoid treatment**

*That is, abnormal thyroid function tests in the 3 months prior to the screening visit.

†Reflecting lactose content of trial medication.

‡Either the woman is planning a pregnancy, or, the woman is at risk of pregnancy and she is not willing to use a non-hormonal method of contraception (condom, diaphragm) until her participation in the study has ended.

§If a patient has discontinued use she may be considered for inclusion in study (provided otherwise eligible), but only after a sufficient wash-out period has elapsed. Required wash-out times are shown in online supplementary table.

¶Immediate release or monthly sustained release depot preparation, or 3 or 6 months sustained release depot preparation.

**Any systemic or inhaled treatment, and/or any ‘potent’ topical, or ‘very potent’ topical preparation (see list in online supplementary box I).

Recruitment (to screening) started on January 2014 and can continue to May 2016, with randomisation up until July 2016.

### Intervention

The treatment regimen will be oral dexamethasone or placebo twice daily, continuing for 5 days, starting on the seventh day after a luteinising hormone (LH) surge has been detected by serial urine dipstick testing (day LHu+7). Thus, for a patient with a regular standard 28-day cycle, treatment should happen on days 20–24, ending 4 days before the start of the expected next period. Women who do not wish to carry out serial dipstick testing will be permitted to participate, but the treatment start date in each cycle will be estimated ‘pro rata’ on the basis of the woman's cycle length documented in previous screening and treatment cycles. Dipstick testing, where used, should start early in the follicular phase (about day 6–9 of a 28-day cycle). In the event no LH surge is detected we will, to avoid missed treatment, specify a ‘latest’ day in the cycle to start treatment, as above.

If a patient is unable to tolerate the trial medication or develops a serious adverse event, or falls pregnant, or starts a prohibited medication (see bottom of box 1), trial medication will be discontinued. The patient will be followed up for safety and efficacy outcomes. Women will be reminded by SMS text message when to start medication. They will also be asked to record study medication intake and return all unused study medication. Any medications which are considered necessary for a patient's welfare, and which are not ‘prohibited’ (see box 1 ), may be given at the discretion of the senior clinical investigator. All concomitant medications taken by the patient during the study from the date of signature of informed consent until the final follow-up visit will be recorded in the appropriate section of the case report form.

### Outcomes

The primary outcome is reduction in mean MBL, objectively measured over the screening and second and third treatment cycles. However, it has been recommended that any interventions should aim also to improve quality-of-life measures.^1^ Secondary indicators of worthwhile improvement will be collected via the Treatment Review Questionnaire completed after third treated cycle, and will address ‘satisfaction’ with treatment (self-reported ‘lighter’ or ‘much lighter bleeding’, and generally feeling ‘much better’ during period), improvement in period pain (‘less’ or ‘much less severe’); and freedom from unacceptable side effects. We will also examine change in menstrual diary score for volume of menstrual period, assessing its reliability and validity as substitute for change in objectively measured MBL.

### Sample size

In trials of medical treatments for HMB it is generally held that a 25% reduction in MBL would be a worthwhile improvement^27^—for example, a 16 mL reduction for an MBL of 65 mL, a fairly typical MBL among women with problem of HMB. The trial had been funded on the expectation that it would be completed within 3 years, and it was expected that recruitment of and completion by 100–110 participants was possible within the shorter time frame for recruitment, to ensure data collection completes about 4 months before the end of funding. Therefore, all simulations took 100 as the total study size and thus 4 patients per month being enrolled. These analyses showed that our adaptive design is estimated to have statistical power of 93.8%, provided within-patient MBL SD is 18 mL and maximum mean MBL benefit over placebo is 16 mL. Patients who withdraw from the study will be replaced, so to have 100 completing the target, enrolment total is estimated at 108, based on prior experience.

### Adaptive randomisation

The adaptive randomisation will proceed in six phases. Across the entire study a fixed proportion of patients (2/7, 29%) will be allocated to placebo, in order to protect the interpretability of the trial results from any drift in participant characteristics during the course of the trial. During phase one (the first 16 patients randomised) the remainder of patients will be assigned to one of the six active dexamethasone daily doses with equal allocation probability. At the end of the first and next four phases the NDLM (Normal Dynamic Linear Model) analysis will be run by the unblinded statistician to ascertain, on the basis of the accumulating MBL primary outcome data, how the randomisation schedule should be adapted. (The adaptation timings have been determined from the pretrial simulations and will take place after 16, 32, 50, 66 and 84 patients have been randomised.) During phases 2–6 the allocation probability for each active dexamethasone dose will depend on the amount of new information that a randomisation would be expected to provide about the underlying dose–response relationship, based on results collected in earlier phases. Specifically, this will be evaluated as the variance of the primary outcome at the current estimate of the ED_95_ (the minimum dose with near-maximal efficacy). The independent data monitoring committee (DMC) will monitor the progression of the adaptive randomisation to verify that the randomisation schedule is being adapted appropriately in response to the accumulating MBL data.

### Sequence generation

Computer-generated random numbers from the uniform (0,1) distribution will form the basis for the allocation sequence. This list of numbers will be generated by the unblinded programmer at Edinburgh Clinical Trials Unit (ECTU) in advance and will be accessed in sequence during the trial to determine the treatment group for the next randomised participant, based on the set of treatment allocation probabilities at the current stage of the adaptive design.

### Allocation concealment

Patients will be randomised via a secure website. Based on the treatment group assigned using the allocation sequence, a medication code from a predetermined ‘reference’ list will be automatically added to a printed prescription. (In a Bayesian adaptive design there is no role for an allocation list, but this ‘reference’ list is needed to ensure concealment.) This prescription will be dispensed by unblinded pharmacist by referring to the reference’ list provided in advance by ECTU, and only the patient number will be included in the treatment capsule bottle labelling.

### Blinding

Unblinded study staff will be the programmer generating the treatment allocation sequence, the pharmacist dispensing the drug (or placebo) using the list of medication codes, the statistician performing the adaptation analyses and generating the DMC reports, the independent statistician validating the DMC reports and members of the independent DMC. Trial participants, care providers, laboratory staff, research nurses and all other members of the trial team will remain blinded to the assigned intervention and regarding adaptations made to randomisation probabilities.

The study drug manufacturer, Tayside Pharmaceuticals^i^ (TP), will manufacture the active and matched placebo capsules for all arms of the trial using dexamethasone micronised powder Ph Eur, Lactose Ph Eur and hard gelatine capsules. (Daily active dose will be split between morning and evening so the 6 strengths of dexamethasone capsules manufactured will be 0.2, 0.4, 0.5, 0.6, 0.75 and 0.9 mg capsules.) The bulk containers will be labelled to identify the strength of the capsules and these will be held (out of sight of any of the research team) by Pharmacy, Royal Infirmary of Edinburgh. Reorder and resupply will be organised between Pharmacy and TP without any involvement by the research team.

### Participant time line

**Figure 1:**
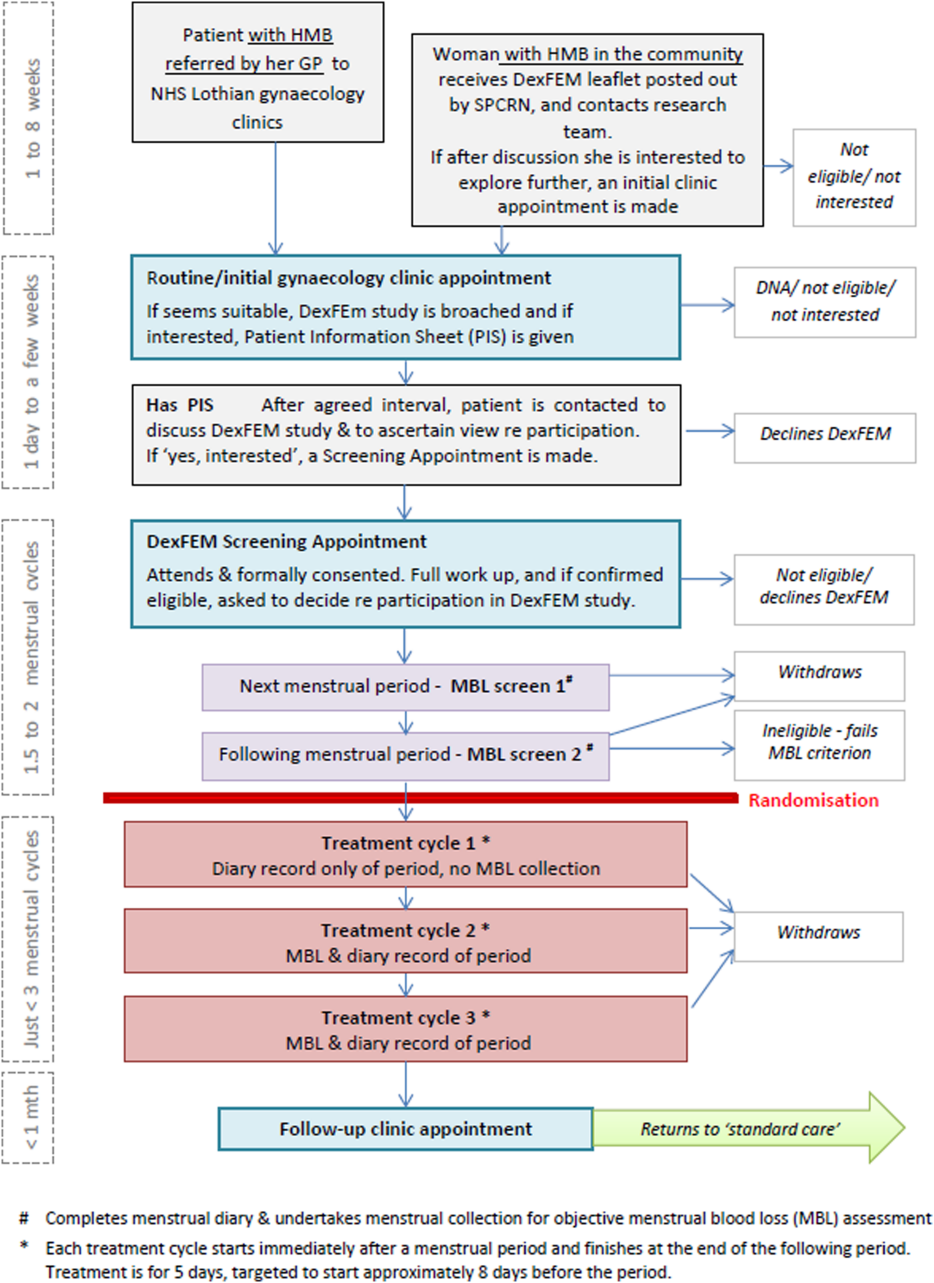
Participant time line(s). *Notes*: #Completes menstrual diary and undertakes menstrual collection for objective MBL assessment. *Each treatment cycle starts immediately after a menstrual period and finishes at the end of the following period. Treatment is for 5 days, targeted to start approximately 8 days before the period (GP, general practitioner; HMB, heavy menstrual bleeding; MBL, menstrual blood loss; mth, month; SPCRN, the Scottish Primary Care Research Network).

Figure 1 shows the patient time line, except it omits an optional clinic appointment that is offered after the first cycle of treatment. From first clinic appointment onwards, any losses to recruitment/retention will revert to standard National Health Service (NHS) care.

### Data collection methods

Table 1 summarises data collection methods and timing. Questionnaires are outlined in the footnotes to table 1—they are based on those used in previous studies.^13^ ^28^ ^29^ The study doctors and in particular study nurses will develop excellent rapport with patients to maximise completeness of data collected. MBL will be assessed by objective laboratory measurement of collected used sanitary protection, will apply modified alkaline haematin method (as previously validated in our laboratory).^28^ ^29^ Assessment of ovarian function, via twice weekly collection of urine samples in third treatment cycle, will allow a check for any effect of dexamethasone on ovarian function.^30^

**Table 1:** Overview of data collection for adaptive trial

			Cycle													
			Screen MBL				Treatment									
			1		2		1			2			3			
Data collection	By whom?	Screen Appt.	M	Post	M	Post	Pre	M	Post	Pre	M	Post	Pre	M	Post	F-up Appt
Consent	Doctor	✓														
Clinical history* (CRF)	Doctor	✓														
Recruitment Questionnaire†	Patient	✓														
Menstrual collection for MBL‡	Patient		✓		✓	✓					✓			✓		
Menstrual diary record§ (screening)	Patient		✓		✓	✓										
Record of dipstick testing for LH¶	Patient						✓			✓			✓			
Study diary (treatment phase)	Patient															
Pregnancy test pretreatment**							✓			✓			✓			
Record of treatment taken††							✓			✓			✓			
Menstrual diary record§								✓			✓			✓		
Uplift MBL collection, for assay	Doctor/nurse			✓		✓						✓			✓	
Check concomitant medications	Doctor/nurse	✓		✓		✓			✓			✓			✓	✓
Check for ‘adverse events’	Doctor/nurse			✓		✓			✓			✓			✓	✓
Ovarian function (urines)‡‡	Patient											✓	✓×6	✓✓	✓	
Safety bloods§§	Doctor/nurse	✓														✓
Treatment Review Questionnaire¶¶	Patient														✓	

*Current health including medication, parity, past treatments etc.

†Detailing HMB problem including duration, associated menstrual symptoms, impact on daily life.

‡See main text.

§To enable an estimate of volume of MBL,^31^ via recording prospectively sanitary product usage/soaking during period, and to elicit, at the end of each period, subjective assessment of ‘heaviness’, menstrual symptoms compared with past 6 months, and any unusual symptoms.

¶(A) If LH testing (by means of commercially available urine dipsticks), date started and date first positive, and (B) whether or not testing, agreed date to start treatment.

**Except for those who have confirmed with clinical team that they are not ‘at risk’ of pregnancy (eg, sterilised, or not in relevant sexual relationship), date of pregnancy test and confirmation negative result.

††Date started medication and confirmation of each morning and evening tablet taken.

‡‡Ovarian function will be assessed by requesting in third treatment cycle collection of twice weekly urine aliquots (to be stored in home freezer compartment until MBL uplift) and subsequently assayed for oestrogen and progesterone metabolites.

§§Undiagnosed (pre) diabetes will be assessed pre-enrolment via plasma glucose and HbA1c, and treatment toxicity will be assessed via plasma glucose, HbA1c, LFTs and urea and electrolytes.

¶¶Subjective assessment of effect of treatment received, in respect of most recent (treated) menstrual period, including comparison of ‘heaviness’ compared with before entering study.

Appt., appointment; CRF, case report form; F-up, follow-up; HbA1c, glycated haemoglobin; LFTs, liver function tests; LH, luteinising hormone; M, menstrual period; MBL, menstrual blood loss; post, as soon as period has ended; pre, run up to period.

### Monitoring

Data on potential side effects will be collected until 30 days after last study tablet; that is, ‘unusual symptoms’ elicited via study diaries and by nurses/doctors during contacts with participants. Expected symptoms in this population are given in online supplementary box II.

The independent DMC convened for the entire project (including the initial small studies) will, for this trial, both review proposed adaptations of the randomisation schedule (see Adaptive randomisation section above), and will regularly review the safety and efficacy data. This DMC will be able to recommend termination of the study in the event of major safety concerns being identified.

## Analysis

### Data management

Trial data accumulation and management is supported by ECTU. Study data will be stored on a secure SQL server at ECTU, the trial database being validated in advance through the use of dummy data and with reference to a documented validation plan. A detailed data management plan will also be held on the secure server at ECTU. Data queries on critical data items will be raised automatically on a monthly basis and circulated to the study researchers for resolution.

### Statistical methods

All analyses will be performed according to the intention to treat principle. A detailed statistical analysis plan, taking a Bayesian approach, will be finalised prior to the locking of the study database and prior to unblinding of the treatment codes.

The dose–response curve for change in MBL between baseline and cycles during randomised treatment will be analysed using a Bayesian NDLM,^26^ which is flexible and requires few assumptions about the shape of the underlying dose–response curve. NDLM will yield considerable efficiency gains, since the estimate of efficacy at a given dose will be informed by that at neighbouring doses. Mean baseline MBL will be included as a covariate in the NDLM. The NDLM analysis will determine which of the doses studied is optimal (in terms of posterior probability of efficacy). For each dexamethasone dose, a 95% credible interval will be calculated for the mean difference in MBL change versus placebo.

Binary or ordinal secondary end points will be analysed using a generalised dynamic linear model. For each dexamethasone dose, a 95% credible interval will be calculated for the OR versus placebo.

No formal stopping rules will be implemented via interim analysis for futility or efficacy. We anticipate minimal missing data on key outcomes and therefore do not plan to use multiple imputation methods to accommodate missing data.

## Ethics and dissemination

### Ethics and governance

The study will be conducted in accordance with the principles of the International Conference on Harmonisation Tripartite Guideline for Good Clinical Practice (ICH GCP).

All women are provided with detailed information about the trial prior to deciding to participate, and provide written informed consent prior to any study-related procedures. Any woman at risk of pregnancy in a treatment cycle will be asked to carry out a pregnancy test as an additional precaution prior to starting the 5 days of study treatment. Patients’ participation is supported by research nurses highly experienced in menstrual research.

Participants can withdraw at any time, or the investigator or care-providing clinician may withdraw the patient if it is deemed medically necessary. The reasons for withdrawal/discontinuation and any adverse events will be recorded. A clear distinction will be made as to whether the patient is withdrawing solely from trial treatments/procedures, or whether the patient is also declining further follow-up, and/or use of data so far collected.

### Dissemination

The trial findings will be made available to participants on request, and will be disseminated via open-access peer-reviewed publications, conferences, clinical networks. All study investigators have a strong and continuing track record in public engagement, and the University of Edinburgh, Queen's Medical Research Institute holds regular public lectures in the “Let's talk…. series”.^ii^

## Discussion

The proposed glucocorticoid treatment, dexamethasone, is an existing, well-characterised drug, widely used in clinical care. Glucocorticoids are even used to treat medical conditions in pregnancy (in first trimester for asthma; rheumatoid arthritis; hyperemesis gravidarum). This means the safety profile is well-known, and the exclusion criteria for this research therefore ‘well-informed’. Furthermore, the modest (short-course) doses being used are periphysiological. (Typical equivalent doses of dexamethasone in an acute exacerbation of obstructive airways disease, eg, would be >3 mg/day, or over 66% more than the maximum dose proposed here.) A further advantage of this phase II ‘new use for an existing drug’ trial, is that if it shows beneficial effect, ‘drug development’ costs for future use will be comparatively low.

The rationale for the use of dexamethasone is derived from mechanistic laboratory studies which suggest a luteal phase glucocorticoid deficiency in the endometrium of women with HMB.^20^ While participants in the trial are not individually assessed to have low levels of endometrial glucocorticoid, it is expected that administration of dexamethasone would reverse local endometrial glucocorticoid deficiency where this is the case.

A unique feature of adaptive trial design is the time lag needed between initiation of research funding and initiating the trial itself, in order to allow the simulation analyses required to develop the design. In this time we undertook two small clinical/mechanistic studies, which involved 15 women in total, and comprised a first use of any modality of glucocorticoid for treatment of HMB. While these studies were not powered to estimate treatment effect, they enabled: collection of safety data (no safety concerns were raised and no patient withdrew during treatment) and mechanistic information (via repeat before-and-after MRI and endometrial biopsies); piloting of methods; and a check on some assumptions that had been fed into the adaptive design simulation model, for example, within-patient variability in MBL. Apart from the opportunity for this preliminary work, adaptive randomisation has notable methodological benefits, in terms of statistical efficiency, and ethically.^24^ ^25^ A disadvantage is that it is not easy to provide a simple explanation of adaptive randomisation in the patient information sheet.

The challenge for this study is recruitment, because a lot is asked. Participation lasts 6 months, comprising screening menstrual collections and diary records for two untreated menstrual periods and then for each of the three treatment menstrual cycles (1) ovulation testing by urine dipstick each morning from about day 8, for about 6–10 days depending on cycle length, (2) taking medication twice a day for 5 days in the mid-luteal phase (exact start date is calculated from ovulation) and (3) diary record of the period. In addition, for the second and third treatment cycles menstrual collection is requested. At start and finish safety bloods are taken and in the last treatment cycle twice-weekly urine collections are requested to assess ovarian function. It speaks volumes about the adverse impact of HMB on women's lives that patients judge it worthwhile to participate in such a trial.

This also underscores the unmet clinical need for a medical treatment for HMB that was noted above, particularly one that is compatible with starting pregnancy. Therefore, demonstration of efficacy with systemic administration of dexamethasone has potential benefit for many women. It will allow further development of this drug as a treatment option, a possible stepping-stone to more sophisticated/targeted steroid treatment of HMB.

## Supplementary Material

Reviewer comments

## Footnotes

i MIA (IMP) 14076) *Tayside Pharmaceuticals|Ninewells Hospital|Dundee|DD1 9SY*.

ii http://www.ed.ac.uk/schools-departments/medicine-vet-medicine/news-events/all-events/new-treatments.
