# Supplementary material for: Low-dose dexamethasone as a treatment for women with heavy menstrual bleeding: protocol for response-adaptive randomised placebo-controlled dose-finding parallel group trial (DexFEM)
Source: BMJ Open. 2015 Jan 14;5(1):e006837. doi: 10.1136/bmjopen-2014-006837 (PMC4298087; doi:10.1136/bmjopen-2014-006837)
Supplement: Reviewer comments [file bmjopen-2014-006837.reviewer_comments.pdf]

PEER REVIEW HISTORY

BMJ Open publishes all reviews undertaken for accepted manuscripts. Reviewers are asked to complete a checklist review form (**http://bmjopen.bmj.com/site/about/resources/checklist.pdf**) and are provided with free text boxes to elaborate on their assessment. These free text comments are reproduced below. Some articles will have been accepted based in part or entirely on reviews undertaken for other BMJ Group journals. These will be reproduced where possible.

ARTICLE DETAILS

**TITLE (PROVISIONAL)**	Low dose dexamethasone as a treatment for women with heavy menstrual bleeding: protocol for response-adaptive randomised placebo-controlled dose-finding parallel group trial (DexFEM)
**AUTHORS**	Warner, Pamela; Weir, Christopher; Hansen, Christian; Douglas, Anne; Madhra, Mayhank; Hillier, Stephen; Walker, Brian; Saunders, Phillipa; Iredale, John; Semple, Scott; Critchley, Hilary

-This article was not externally reviewed at BMJ Open. Protocols that have been independently assessed prior to submission to BMJ Open are usually fast-tracked to publication on the grounds that further substantial changes will not be possible. This independent assessment will usually be external, independent review for both a substantial grant award from a non commercial or government funder and ethics approval.
